# A Modular Digital Twinning Framework for Safety Assurance of Collaborative Robotics

**DOI:** 10.3389/frobt.2021.758099

**Published:** 2021-12-10

**Authors:** J.A. Douthwaite, B. Lesage, M. Gleirscher, R. Calinescu, J. M. Aitken, R. Alexander, J. Law

**Affiliations:** ^1^ Department of Automatic Control and Systems Engineering, University of Sheffield, Sheffield, United Kingdom; ^2^ Department of Computer Science, University of York, York, United Kingdom; ^3^ Mathematics and Computer Science, University of Bremen, Bremen, Germany; ^4^ Department of Computer Science and the Advanced Manufacturing Research Centre, University of Sheffield, Sheffield, United Kingdom

**Keywords:** collaborative robot safety, digital twins, modular framework, risk-informed software synthesis, probabilistic model checking, automated code generation, human-robot collaboration, robotics

## Abstract

Digital twins offer a unique opportunity to design, test, deploy, monitor, and control real-world robotic processes. In this paper we present a novel, modular digital twinning framework developed for the investigation of safety within collaborative robotic manufacturing processes. The modular architecture supports scalable representations of user-defined cyber-physical environments, and tools for safety analysis and control. This versatile research tool facilitates the creation of mixed environments of Digital Models, Digital Shadows, and Digital Twins, whilst standardising communication and physical system representation across different hardware platforms. The framework is demonstrated as applied to an industrial case-study focused on the safety assurance of a collaborative robotic manufacturing process. We describe the creation of a digital twin scenario, consisting of individual digital twins of entities in the manufacturing case study, and the application of a synthesised safety controller from our wider work. We show how the framework is able to provide adequate evidence to virtually assess safety claims made against the safety controller using a supporting validation module and testing strategy. The implementation, evidence and safety investigation is presented and discussed, raising exciting possibilities for the use of digital twins in robotic safety assurance.

## 1 Introduction

Collaborative robots promise to transform the manufacturing sector, enabling humans and robots to work together in shared spaces and physically interact to maximise the benefits of both manual and robotic processes. As such the market for collaborative robots has seen rapid growth in recent years, and is predicted to reach $5.6bn by 2027, accounting for 30% of the total robot market (Interact analysis, 2019). However, in practice safety remains a critical issue and a barrier to truly collaborative processes: international standards (ISO/TS 15066, 2016; ISO/TS 10218, 2011a; ISO/TS 10218, 2011b) provide requirements for safe operation, but meeting these in complex settings is difficult and there is little guidance on how to develop safe collaborative processes. In the United States, NIST have identified key barriers to human robot collaboration including ensuring “safe human robot interaction”, providing accurate “sensing and perception for unstructured environments”, and providing realistic “modelling and simulation” (NIST, 2016).

**FIGURE 1:**
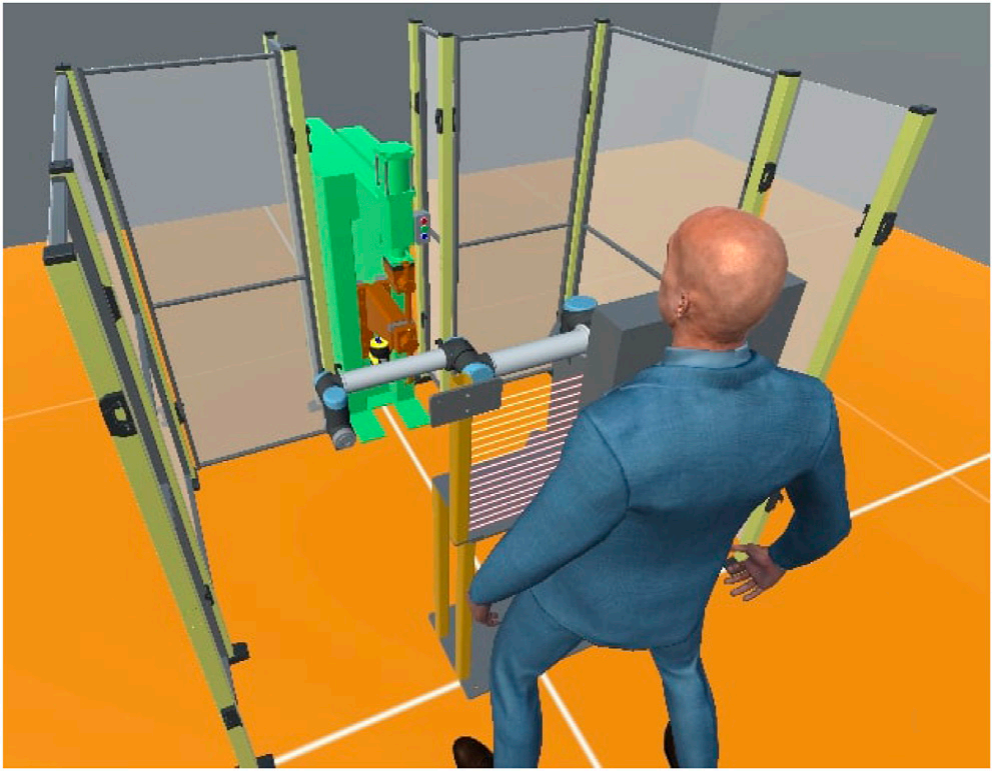
An isometric view of the CSI framework environment applied to a collaborative welding cell case-study. Based in Unity^®^ 3D, digital twins of an operator, collaborative robot manipulator, safety scanner and light-barrier are shown.

Our work on the Confident Safety Integration for Collaborative Robotics (CSI:Cobot) project (here after referred to as CSI) is addressing these challenges and developing techniques for the safety assurance for industrial collaborative robotics^1^. Due to Covid-19 restrictions limiting access to physical facilities in recent times, we have adopted the novel approach of using digital twins (DTs) to support our research. Specifically, we have developed a modular, general-purpose DT framework (shown in Figure 1) which enables on-line and off-line development, testing, deployment, and validation of safety tools. This has opened exciting new possibilities for robotics safety research, and is underpinning our activities into safety sensing and decision making, safety testing, controller synthesis, cyber security, and safety visualisation (Wang et al., 2021; Gleirscher and Calinescu, 2021; Lesage and Alexander, 2020; Gleirscher et al., 2021; Lesage and Alexander, 2021).

Digital twins present an opportunity to gain unprecedented access to a manufacturing process and its data in order to interrogate, monitor and control physical assets and processes in real-time and throughout their life cycle. They have been cited as a key enabling technology for Industry 4.0, enhancing the information available to process engineers, safety engineers and management and supporting the rapid design, simulation, testing, verification, deployment of future systems (Bolton et al., 2018; Agnusdei et al., 2021b). In particular they allow critical “what if?” and “why?” questions to be answered within the design cycle, and the exploration of system choices and their impact on operability and deployment.

We have developed a modular DT framework that takes advantage of the above properties, and allows us to combine simulated and real-world data to assess the safety of collaborative robotic processes both during the design phase and during operation. The modular nature of the framework has been crucial to the development of DTs that represent multiple physical systems with variable capabilities, limitations, and complex relationships with external hardware. Modularisation allows this complexity to be managed systemically and invoked to create sophisticated, realistic case-study scenarios. Specialist tools developed within the framework allow investigation of topics including risk and mitigation strategies, which can be rapidly redeployed (or extended) in response to changes in configurations or conditions as processes are developed. Furthermore, the nature of digital twins enables safety techniques developed in the digital domain to be deployed in the physical domain using the same environment.

In the remainder of this article we present our modular framework and demonstrate its application to the safety assurance of a collaborative robot spot-welding process. We begin in Section 2 by introducing the concept of a safety-critical digital twin, followed by a review of related work in Section 3 and the case for a new modular approach to support safety assurance. In Section 4 we describe our framework, and the modular representation used in detail. In Section 5 we demonstrate its use in assuring the safety of an industrial case study focusing on two modules: 1) an experimental safety controller, and 2) an evaluation module used validate that controller. The results of the case-study are presented and discussed in Section 6 with conclusions and future development discussed in Section 7.

## 2 Background

### 2.1 What Is a Digital Twin?

In the growing body of literature, the term *Digital Twin* (DT) has been used inconsistently to define different relationships between the physical and digital components. In Kritzinger et al. (2018), an in-depth review is categorising concepts associated with DTs is provided. Concepts such as the *Digital Shadow* (DS) and *Digital Model* (DM), differ with respect to the communication infrastructure between the physical and digital components; which is typically unilateral or not considered, respectively. A clear definition of a DT can, however, be found in Grieves and Vickers (2016) as:

“a digital informational construct about a physical system … created as an entity on its own. This digital information would be a “twin” of the information that was embedded within the physical system itself and be linked with that physical system through the entire lifecycle of the system.” (Grieves and Vickers, 2016)

A DT could then be said to take the level of integration further, in that information regarding the state of the physical and digital components can be interrogated simultaneously and compared. The user is presented with a single interface to the *cyber-physical system* (CPS) that persists throughout its operational life time (Kritzinger et al., 2018; Qi et al., 2018).

### 2.2 Why Digital Twins?

DTs represent a fundamental change in the relationship between conventional simulation approaches and the associated hardware. At its core, these differences can be broken down into three key areas:

1. **Persistence**—A DT is virtual record of a physical system’s life-cycle. Information on the individual system’s (i.e., usage or deterioration) can captured regularly, modelled stored and maintained across applications.
2. **Insight**—Similar to conventional simulation, DTs can be used to predict *what may* happen on their system. DTs however provide an interface to *what is* happening on the hardware now and provide a means to interrogate the design.
3. **Explainability**—Traditionally, simulations cannot be used to provide insight into the interactions of physical systems; instead depending on accurate understanding of the possible *what-if?* scenarios. DTs present a unique insight for investigation into unexpected behaviours when they occur and diagnose points of failure. This provides evidence to support process safety, design and inform future simulations.

System statistics can be simultaneously extracted, compared and analysed to better understand and respond to divergences between simulation (expectations) and reality (feedback). This is crucial in scenarios where real-time responses must be enacted to avoid unsafe conditions as they develop.

Well established, versatile DTs present a number of opportunities to enhance collaborative safety in manufacturing environments, which can be classified into the following key areas:

1. **Validation**—A DT can provide a virtual interface to a real-world physical process as a means to validate and compare actions across the component’s life-cycle. Scenarios that may be expensive, time-consuming or too dangerous to reproduced physically can represented with a DT and evaluated against operational claims and constraints.
2. **Analysis**—High fidelity DTs interacting with a physical twin (PT) in dangerous scenarios can provide additional sources of structured data independently of the capabilities of the standard PT. This source is extensible, customised and structured and presents a hybrid view of the digital and physical domains. Storage of such information presents a unique opportunity for forensic analysis and diagnostics.
3. **Prediction**—A live DT is in a unique position to form predictions ahead of the current PT state. Actions of the PT can be previewed in light of defined safety constraints, and its response to anomalies assessed, ahead of any physical constraint violation. State-of-the-art models of the component allow performance projections to be generated (for example interference, degradation and damage).
4. **Enhancement**—The duality of the PT and DT provides a scalable interface for expanding the base functionality of the PT. Parallel modelling of the PT using a DT facilitates a better understanding of anomalous behaviour; this may be the result of a malicious action, component failure or procedure violation. Using the additional feedback of a DT (and additional DT sensors), proprietary systems can enact executive decisions in a wider information space.

Until recently, DTs have been typically operated as closed systems emphasising extended functionality of an individual system or robot. As a result, the advantages of wider data access in systems of multiple DTs have been largely neglected. Modular frameworks that allow the fusion of system-level DTs into one connected *scenario* DT (a connected ecosystem of individual DTs) create a feasible way to observe these benefits and access multi-level data for greater process insight and situational awareness.

### 2.3 Industrial Safety in Robotic Systems

In domains such as robotic manufacturing or passenger road vehicles, the certification of safety-critical systems and parts thereof is regulated by standards, such as ISO/TS 15066 (2016) and ISO 26262 (2011). These standards make recommendations about how safety assurance is to be carried through and what is to be delivered to the relevant certification authorities, typically in the form of what is called a *safety* or *assurance case*.

A *safety case* delivers a structured and *evidence*-based *argument* for the *claim* that a system is safe to be operated in a particular context or in certain use cases (McDermid, 1994). It is a result of assurance tasks that deal with the creation and combination of the said evidence. Before a given process can be commissioned, compliance with the claims must be evidenced to demonstrate that “due diligence” has been performed.

Digital twins provide a unique opportunity to generate evidence to support a safety case and the verification of a systems compliance to the given standards. They create an interface to a process that allow safety engineers and control engineers to define testable process constraints and predicates to validate claims made against the twin’s properties and assumptions (i.e., geometry, programming, tools and usage limits).

## 3 Related Work

Our work builds upon several established concepts within the digital twin community, and those of automated testing and industrial automation. While the DT concept has been around since the early 2000s (Glaessgen and Stargel, 2012), literary examples of DTs are typically specific in application and narrow in focus (Damjanovic-Behrendt and Behrendt, 2019; Agnusdei et al., 2021b).

More recently, increasing interest in DTs and associated technologies has brought more clarity on the challenges associated with generalising DT approaches, requirements, safety and supporting standards on the path to general purpose solutions (Bolton et al., 2018). Reviews examining the literary distinctions and terminology for technologies associated with DTs can be found in Kritzinger et al. (2018), Jones et al. (2020). Surveys examining these standards and requirements can be found in Jones et al. (2020), Lu et al. (2020), Agnusdei et al. (2021b), Hou et al. (2021). Discussions on approaches and cross-application needs are also found in Mabkhot et al. (2018), Bolton et al. (2018), Agnusdei et al. (2021a) with varied definition of the term “digital twin”. This step towards more flexible, general purpose DTs will be crucial as we create more automated, connected and situationally-aware production environments and increasly transfer domain research and knowledge (Mabkhot et al., 2018; Damjanovic-Behrendt and Behrendt, 2019; Lu et al., 2020).

In Guo et al. (2019), an entity-modular approach to flexible digital twin definition is proposed as a mechanism of increasing factory layout efficiency. Twin’s are encapsulated in discrete modules, as low fidelity models of their physical counterparts. A entity interface is presented as a standardisation technique for a collection of DTs and enabling communication between distinct models. The approach is shown to increase machine up time and shop floor mobility by using DTs to inform models of many factory floor designs simultaneously. The authors employ application-specific software (Siemens Factory Planner) so the transferability of the approach is limited. Tao and Zhang (2017) present the notion of a digital twinned shop floor. Here, several challenges facing the implementation of DT-based smart manufacturing are outlined in relation to data management, service tendering and interfacing with physical systems. In Qi et al. (2018), DTs are discussed from the perspective of service brokering with a DT shop floor. XML is proposed as a communal language describing the capabilities, parameters and service viability in a generalised service manager.

In Shahsavari et al. (2021), an open-source architecture “Model Conductor-eXtended” is proposed. A simulated drone is used to demonstrate communication with the framework *via* a unilateral Message Queuing Telemetry Transport (MQTT) protocol. The current limitations of their framework indicate that their models are more appropriately labelled digital shadows as full bilateral data exchange with a physical twin is not demonstrated (Kritzinger et al., 2018). Zhou et al. (2020) present a similar concept of a knowledge-driven digital twin manufacturing cell which utilises a MQTT broker to enable bilateral communication between a PT work cell and its corresponding DT. The need for stable, uniform system interfaces for communication between the DT and PT is highlighted, emphasising the challenges resulting from the lack of synchronisation techniques and “out of step” data. These challenges are also highlighted in a recent review (Lu et al., 2020), emphasising the need for standardisation (with respect to communication mechanisms, model representation and protocols), based on existing industrial standards where appropriate.

Masison et al. (2021) present a framework to support collaboration and distributed modelling of medical processes using a modular design pattern. The example given centres on infection modelling in the respiratory system and their results are compared to a conventional simulation model. While the approach is demonstrated to be more efficient, scalable and present a lower skill barrier to users, the authors do not incorporate feedback from the physical system. The results presented in these works are indicative of successful implementation of a distributed digital model, rather than a digital twin (Kritzinger et al., 2018). In Bohlin et al. (2017) the authors proposes a generic approach to real-time geometry assurance in the realisation of their “Smart Assembly 4.0”. Their framework is demonstrated on a spot welding process and rendered as a 3D visualisation, with an Apache Kafka interface under development and proposal to use ROS to expand the capabilities of the platform.

Many of the challenges toward the development of DTs for collaborative industrial processes have been discussed historically, with varying terminology and fidelity. It is clear there is a need for frameworks that are both able to create easy access to complex DT definitions and behaviours, and standardise their application and terminology (by appealing to modern industry standards/protocols). The literature has shown that conventional approaches are typically narrow in application. Frameworks that allow the generalisation of DT concepts using a versatile, scalable approach would therefore be invaluable moving forward into industry 4.0 and the future of digital manufacturing.

The versatile modular framework presented in the following sections provides the necessary infrastructure to facilitate many of the demands outlined in the literature. Based in Unity®, a complete environmental package is proposed that enables reconstruction of complex cyber-physical processes. The software trivialises the creation of advanced modelling concepts, DT behaviours and utilities to a “drop in place” interaction within an attractive 3D world—where the results are visualised. We examine how users are able deploy new modules to create custom investigations/control schemes using a case study involving safety assurance of a collaborative manufacturing process. Two example modules are described, deployed and used to evaluate the coverage of the proposed controller and the resultant safety of the real-world process using the modular digital twin framework.

## 4 The CSI Digital Twin Framework

In this section we present the modular digital-twinning framework ^2^ developed as part of the CSI project. The CSI framework was developed to support our broader objectives of investigating safety and security assurance within cyber-physical systems (CPS) and collaborative robot processes. This involved the development of a tool able to support a wide range of safety topics, interactions and scenarios so that their safety may be investigated and evidenced.

The CSI framework, as a result, is a versatile, modular tool that can be used to assemble safety-critical digital twins with variable levels of complexity and fidelity. Using the commercial game engine Unity^®^ as base, it facilitates advanced visuals, basic physics simulation as well as connections to modern AR/VR peripherals and utilities (see in Figure 1). Presentation of digital twins as a collection of modular systems and behaviours allows the user to represent a variety of processes, safety cases and case-studies using an architecture that is principally developed and informed by safety.

### 4.1 Modular Representation

**FIGURE 2:**
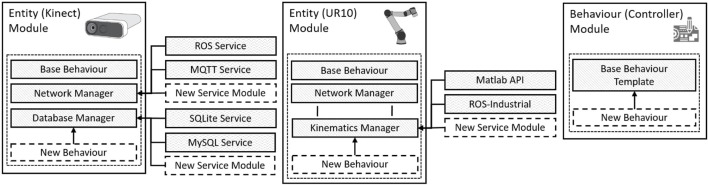
An overview of the relationships between *entity* modules, *behaviour* modules and *service* modules. Entity modules may invoke service or behaviour modules as part of their behavioural “stack”. The composition of the stack is an aggregation of custom behaviours and inherited modules from broader entity classifications.

In the description of arbitrary CPS (i.e., a manufacturing processes), with an arbitrary number of sensors, manipulators and operators, we define three initial concepts. 1) *entity*—modules with a distinct collection of models, state-machines and behaviours that emulate or interface with the capabilities of the physical twin, 2) *behaviour*—modules without physical embodiment that may introduce unique programming, or control or communicate with other entities, and 3) *service*—modules without physical embodiment that may provide interfaces to specialist toolboxes or utilities. As shown in Figure 2, these concepts are defined as distinct *module types* based on their function within the CSI framework.

#### 4.1.1 Entity Module Representation

Individual DTs are presented in the form of *entity* modules with behaviours associated with providing a simulated avatar (digital components) and information sources from the corresponding real device (physical components). When an entity is not associated with a *physical* twin (PT) (no associated hardware or connection(s)), a conventional simulation interface can be upheld using the entities standalone *digital* behaviours. The aggregation of which is referred to as the entity’s *digital* twin (DT).

Digital twins become distinct from convention simulation tools when real-world systems and data sources are configured to interact with an entity module. Here, an entity module represents and unique relationship with a specific device or hardware system based in the real-world. The behaviours associated with managing that relationship are attributed to the entity’s physical behaviours and *physical* twin (PT). The nature of the interaction between a DT and its corresponding PT is dependent on the capabilities of the PT, but may also vary significantly between use-cases. The DT and PT behaviour modules collectively define the entity’s *behaviour stack*; as both the digital representation of the system (a means to simulate it) and it’s physical representation (a means to interact with it physically). This provides a basis to characterise a functional digital twin, its capabilities as a CPS, and create a means extend existing definitions with further behaviours, connections and interfaces.

The CSI framework distributes these entity behaviours in the form of behaviour modules based on the entity’s high-level *classification* (i.e. robot, sensor etc) and concepts of inheritance^3^. Each entity is characterised by it’s behavioural “stack” (see Figure 2), containing the aggregate capabilities and defaults of broader classifications (i.e. device, human). As a result, complex system representations can be made available to the user by default, including I/O control, state-machine representations, communication and service interfaces, without the user explicitly implementing them. This default behavioural stack of any entity module may then be extended to include additional controllers, analysis tools or behaviour modules.

We define a *scenario* DT as a unique collection of modules, their configuration, and relationships with the an external process. Scenario DTs represent a multi-level interface to the physical process, in which high-level data (such as performance, control and state information), and low-level data (component health signals) may be interrogated. This provides a versatile, holistic, view of the process, its individual constituent modules and behaviours that can inform the user on the wider safety context within their collaborative process.

The versatility of the CSI framework becomes more evident when the DT behaviour inheritance scheme is combined with the notion of twin-mode switching. These modes are defined based on three distinct principles relating to the direction of information exchanged with the PT (Kritzinger et al., 2018). The corresponding *twin-modes* representing these groups are defined as follows:

•**Model**—A digital model (DM) has no exchange of state data with a PT system. This mode provides a minimal representation of a DT within the framework. This may be considered akin to classical simulation of the system, with interactions between this entity and other entities defined as uniquely virtual.
•**Shadow**—A digital shadow (DS) represents a unilateral exchange of state data from the PT to the framework. The entity is presented as a *hologram* of the PT, and may considered analogous to some principles of hardware in the loop (HiL) simulation. This allows the state of the PT to be observed and displayed within the framework.
•**Twin**—A full digital twin (DT) captures bilateral state data exchange with a target PT. Dual representation of the DT and PT allows the state of both systems to be observed and visualised simultaneously.

The functionality of any one entity module (or entity DT) is therefore discerned by the active *twin-mode*. The notion of twin-mode switching is a powerful feature of the CSI framework as it allows the user to fluidly represent DTs, DSs, DMs and their corresponding configurations as a single entity module, without modifying the design of the process (Bolton et al., 2018; Kritzinger et al., 2018).

Consider the example where a new system is to be introduced into an existing scenario DT that represents the user’s current process. Their scenario is complex, containing a number of connected entities in the form of DMs, DTs and DSs and the user wishes to investigate the safety implications of the new system ahead of investment. As the system is not available, no data can be provided to the CSI framework from the PT (as a DS or DT), and so the system is introduced to the scenario as a DM, to allow the response of the other modules to be observed safely, and the precise configuration (positioning, tool usage) analysed and explored.

Once the revised scenario configuration is demonstrated, the user invests in the new system and it is installed. Initially, the new system can be represented as a DS which provides accurate real-time telemetry of the PT to the entity module. This provides awareness to neighbouring DTs in the process with increasing fidelity as more features of the PT come online. As the level of integration of the new system increases, the relationship between the PT and the entity module can seamlessly transition to a complete DT representation. Once the module is in DT mode, state data from both the DT and the PT can be used to inform high-level logic and decision making processes about the desired state of the process. The use of twin-modes creates a “plug and play” paradigm that allows new process configurations to be evaluated and compatibility issues to be identified before investing in a specific hardware platform (Dennis et al., 2014; Aitken et al., 2014; Ippolito et al., 2005).

#### 4.1.2 Behaviour Module Representation

Behaviours not directly representing a PT system (such as process controllers, analyses, or AI elements) are represented as *behaviour* modules. These modules provide a general purpose template defining only core communication and environmental interfaces. While they may exist independently, such modules are typically added to another standard entity DT module to facilitate more complex individual behaviours as shown in Figure 2. This may be achieved by implementing additional logic, message broadcasters or receivers in order to extend the behaviour of another module.

Industrial process controllers are defined as behaviour modules within the environment, containing references to their subordinate modules. Messages originating from one module are received by entities subscribing to that control signal and enact a response dictated by the receiving module. Examples of complex controller integration into the CSI framework can be found in related works (Gleirscher and Calinescu, 2020; Gleirscher et al., 2021).

#### 4.1.3 Service Module Representation

To support entity and behaviour module definitions, *service* modules are employed to allow entities and behaviours to invoke specialist services and utilities as shown in Figure 2. The service provided by a service module may be handled by framework-native functionality, or *via* an interface to an external network location, toolbox or *Application Programming Interface* (API).

Entity modules interact with service modules through *service managers*. These enforce a standardised interface on their service modules to create a “plug and play” topology and ensure modularity and compatibility across different configurations. A hierarchical design pattern is used to enforce further requirements on more specialised service classifications. This may include any necessary conversions (i.e mapping between coordinate frames), or translation protocols for an external tool (or proprietary language) to integrate it with the communal environment. Isolating the toolbox-specific logic allows the toolbox to operate under the principle of *functionality as a service* (Bolton et al., 2018) whilst enabling the development of more advanced service modules in the future (Qi et al., 2018).

The interaction between entity modules and service modules is necessarily time-variant due to the concept of twin-mode switching and runtime configuration changes. A modular service architecture is, however, well suited to diverse arrangements of entities as a relationship between an entity and a service may be defined in the behaviour manager and simply brokered from an available service module. This allows new service/interface relationships to be created dynamically, under a distributed topology, as the service provider exists distinctly from the invoking entity module.

#### 4.1.4 Example: Modelling a Human Operator

Human operator twins can themselves may be represented as entity modules. Similar to other entity models, a human may be abstracted into a dual digital/physical component representation. The digital component is defined by a basic motion/animation modules to provide parameterisation of the model. This base model is then coupled with an additional programmable behaviour module that provides basic movement autonomy and path-planning. This high-level representation provides a basic interface for simulating the nominal behaviour of a human actor in the user’s scenario. It also provides a means to investigate safety of the operator as the nominal condition evolves (i.e., observable risks or collisions). Within the CSI framework human models can dropped into the scenario and parameterised to begin simulating collaborative processes.

The physical component of the human-entity module provides a convenient interface for introducing user telemetry data into the scenario, informed by wearable devices and sensors positioned on the real operator (or virtual reality peripherals (see Section 4.2.4). This would allow the response of an active process to be observed as a result of a real “human in the loop” interaction, in a safe and controlled environment, when true human decision-making would be difficult to simulate.

### 4.2 Core Systems

**FIGURE 3:**
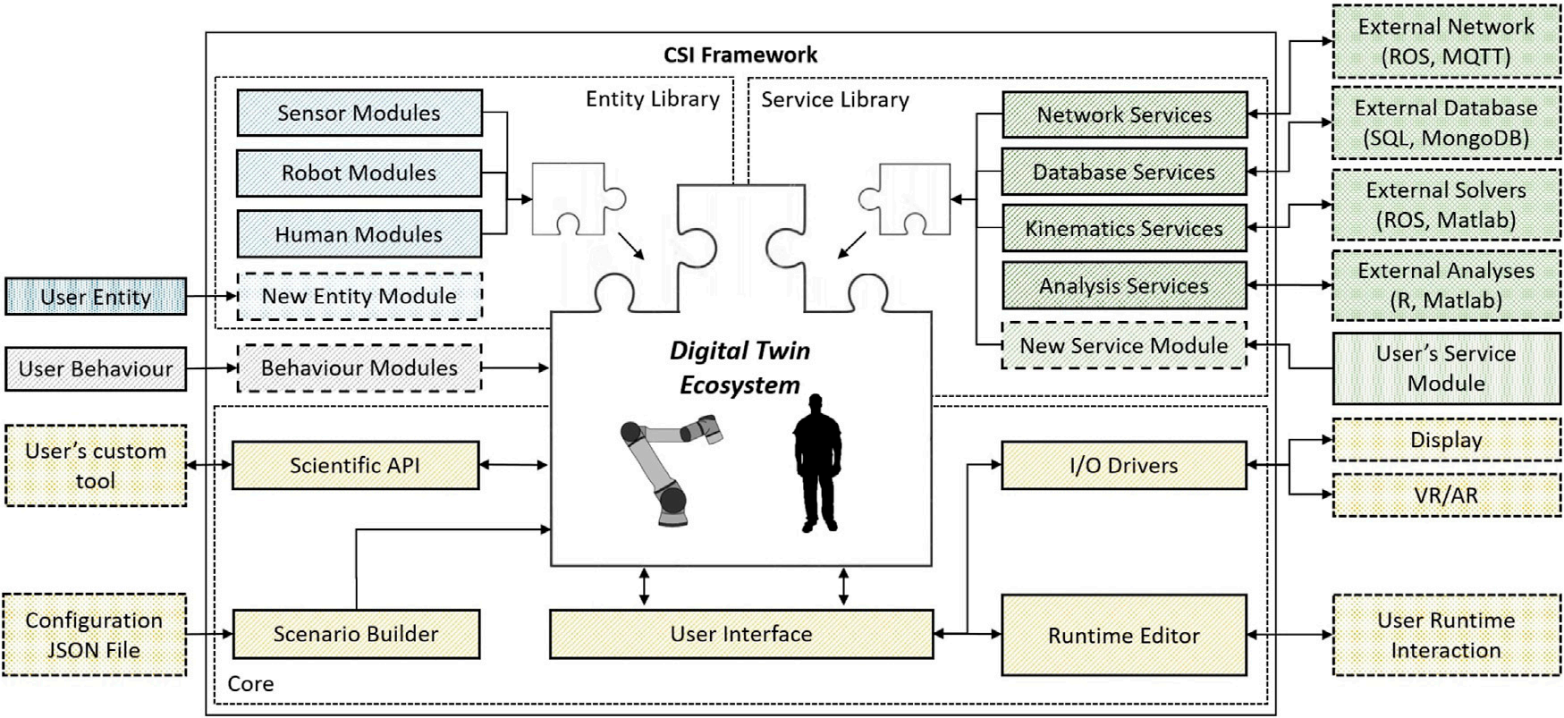
An overview of the CSI modular digital twin framework, entity (blue), service (green) module libraries, behaviour (grey) modules and their integration with the core framework (yellow). User configured modules and extensions to the standard environment are shown as dashed.

The composition of modules is created by the user during the design of their scenario DT. The modularity of the environment is enabled by a versatile core architecture shown in Figure 3.

#### 4.2.1 Communication and Control

Communication between modules inside the CSI framework is handled through a publish-subscribe messaging pattern^4^. This versatile pattern allows the representation of multiple topologies: one-to-one, one-to-many, or many-to-one (Aitken et al., 2014). The modules, as publishers, subscribers or both, do not need to be concerned with the source or destination of their message and only define the communication channel they use. System and environmental events are broadcast similarly, and may be accessed in the form of messages to subscribing modules. This provides modules with a source of ground truth data for analysis (i.e., collisions, damage or connection updates).

Outside-facing data streams, between the framework and external platforms, are managed by the service modules as shown in Figures 2, 3. Service modules expose within the CSI framework an interface defined using the same publication-subscription abstraction. They thus abstract the specific database languages (MySQL, SQLite etc.) or network protocol (TCP, UDP, etc.) from the entity module. A critical aspect of digital twinning is the communication between the DT and its target PT. As standard, all network-facing DT entity modules invoke a network service manager as a fundamental behaviour facilitating communication with their PT. The network service manager provides the interface through which the broadcasters and receivers communicate with an outside network, the directionality of which is determined by the active twin-mode (see Section 4.1.1).

The user is then able to connect the array of systems to a common *Local Area Network* (LAN) and load their associated middleware to expose their communication streams. Within the active CSI framework, the user simply provides the protocol (TCP, UDP, etc.), connection information (such as the IP and port) and any necessary credentials to the DT module which retains this information as a “profile” of its PT’s requirements.

#### 4.2.2 Example Connection With the Robot Operating System

An example of a network service module developed for the CSI framework is the ROS service module. This extension module provides access to the open-source ROS network/protocols and packages as middleware for a number of commercially available robotic platforms and sensors. Together with ROS-industrial, this provides a skeleton infrastructure directly compatible with the CSI framework.

Modules defining an entity DT are dependant on network services to communicate with the target PT and so may invoke this service in accordance with its active twin-mode (see Section 4.1.1). In the event the PT is involved, new network service relationships are dynamically created. Here, defined channels are made available to broadcast or subscribe to/from the associated PT system. This allows the DT entity to be informed by changes in the PT’s state, such as events or joint-states, by associating a channel with the appropriate ROS topic. The CSI framework library includes most standard ROS message definitions (e.g.moveit) and, when communicating with a ROS network, requires minimal conversion.

Use of ROS as the initial middleware platform for PTs immediately allows a diverse range of research and industrial systems to communicate with the environment with only minor configurational changes. Furthermore, it estblishes the basis for future service modules such as moveit2 and ROS2 (anticipated to be distinctly more robust protocols for robot communication).

#### 4.2.3 Workflow

**FIGURE 4:**
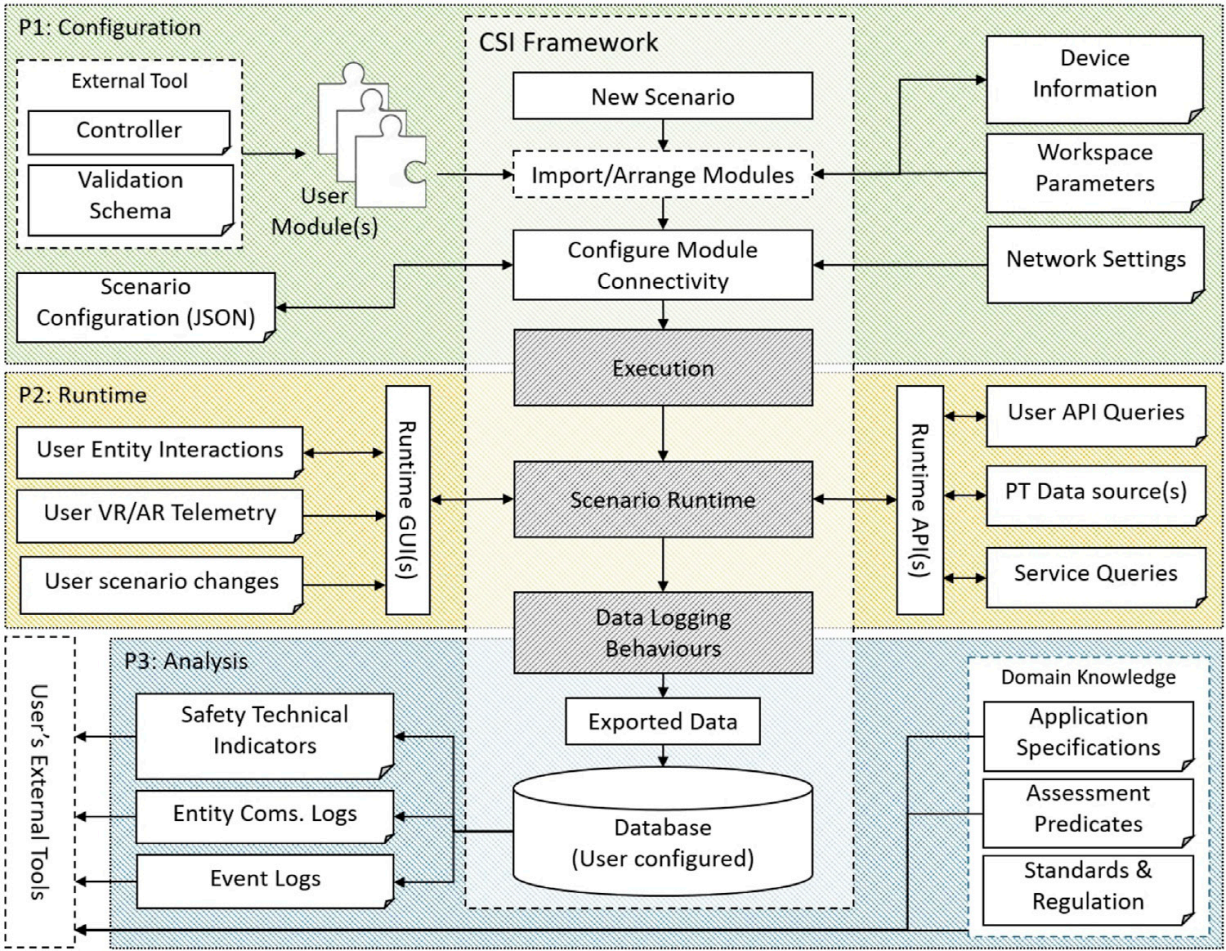
An overview of the CSI framework workflow. Initially (P1), the user creates their scenario, modules and configures them. In the execution phase (P2), the scenario is processed and may be interacted with during runtime. During runtime, configured logging behaviours write to the user’s database(s) (i.e., SQLite, MySQL). This log data is parsed and imported in the Analysis phase (P3). A provided database API provides convenient interface for the users external tools.

The CSI framework presents two distinct operational modes, namely; 1) *Online*—As a live interface for control and safety monitoring overseeing an active process, or 2) *Offline*—As a faithful process test-bed for simulation-based analysis. An overview of the user’s interaction with the software is described *via* the workflow presented in Figure 4.

The user assembles their scenario DT using the configuration tools and utilities. This may involve importing unique user module definitions or simply selecting from a library of existing module definitions. The user configures their modules with connection information, communication channels and associates control regimes with subordinate entities to define their nominal process. Additional modules may be introduced to expose further data streams for analysis or augmentation. The module configuration may then be exported using the *scenario builder*; the same integrated *JSON* import/export tool is executed from within the runtime environment to prepare, or save changes to, the scenario’s configuration.

In the event the CSI framework is used as a simulation test-bed, the environment may be operated headless and executed programmatically as an executable file. In this mode, the scenario configuration may provided as part of an external process and imported using the *scenario builder* as shown in Figure 3. This provides an interface for augmenting the scenario’s initial conditions programmatically or specify exit conditions to be able to facilitate exploratory analyses (see Section 5.4). This might be in the form of mutation testing or Monte Carlo Analysis, where the DT’s response to many configuration changes is observed and analysed.

At runtime, information from the scenario such as events, collisions and notifications are stored in a database. The user may choose to see and interact with the scenario through the provided GUI, shown in Figure 1, or operate in headless mode for optimal performance. Twins with database behaviours are able to archive custom data streams for targeted analyses (see Figure 2). This provides the user with the flexibility to generate a historical record of their own key performance indicators. At the point of analysis, the user is presented with a structured data archive, or *black-box*, representing the scenario history which may then be interrogated using external tools.

#### 4.2.4 Virtual and Augmented Reality

Being able to interact with the PT for the purpose of manual operation, inspection, or diagnostics allows the operator to familiarise themselves with its functionality and problems that might have occurred. With Unity^®^ 3D at the base of the CSI framework, detailed robotic processes can be similarly visualised and expose detailed diagnostic information about the state and health of entities (PT and DT) simultaneously. Using our interface for Microsoft’s Mixed Reality Toolkit (and others), this relationship between the PT, DT and the operator is further enhanced through the use of *Virtual Reality* (VR) and *Augmented Reality* (AR) (as shown in Figure 3).

AR/VR peripherals and other wearable sensors also provide a means to inform operator models as digital avatars where, from the perspective of the operator, demonstration of the dynamic response of an ecosystem of VR/AR twins provides a distinctly richer and intuitive experience. Human modelling as entities may be seen in Section 4.1.4. In the literature, previous DT technologies emphasis the availability of DT information using primitive tools or command-line utilities, with only recent works starting to utilise VR/AR (Karadeniz et al., 2019; Zhu et al., 2019). These examples however are strictly limited to a given use case, with standards and generalised DT utilities not considered. Creating a standardised interaction with DTs in VR/AR presents a number of opportunities in the areas of safety visualisation, operator training and remote operation using the CSI framework.

## 5 Case Study—A Real World Industrial Process

In this section a real-world industrial manufacturing process at a plant in the United Kingdom is presented as a demonstrative case-study. The example presents a collaborative robotics scenario with multiple collaborating DTs hosted by the modular framework. The modular nature of the framework allows multiple components to be combined to represent once holistic scenario. This allows the complexities and associated risks of a modelled system to be associated with the individual modules and used to isolate novel safety concerns in new scenarios.

### 5.1 The Collaborative Welding Cell

**FIGURE 5:**
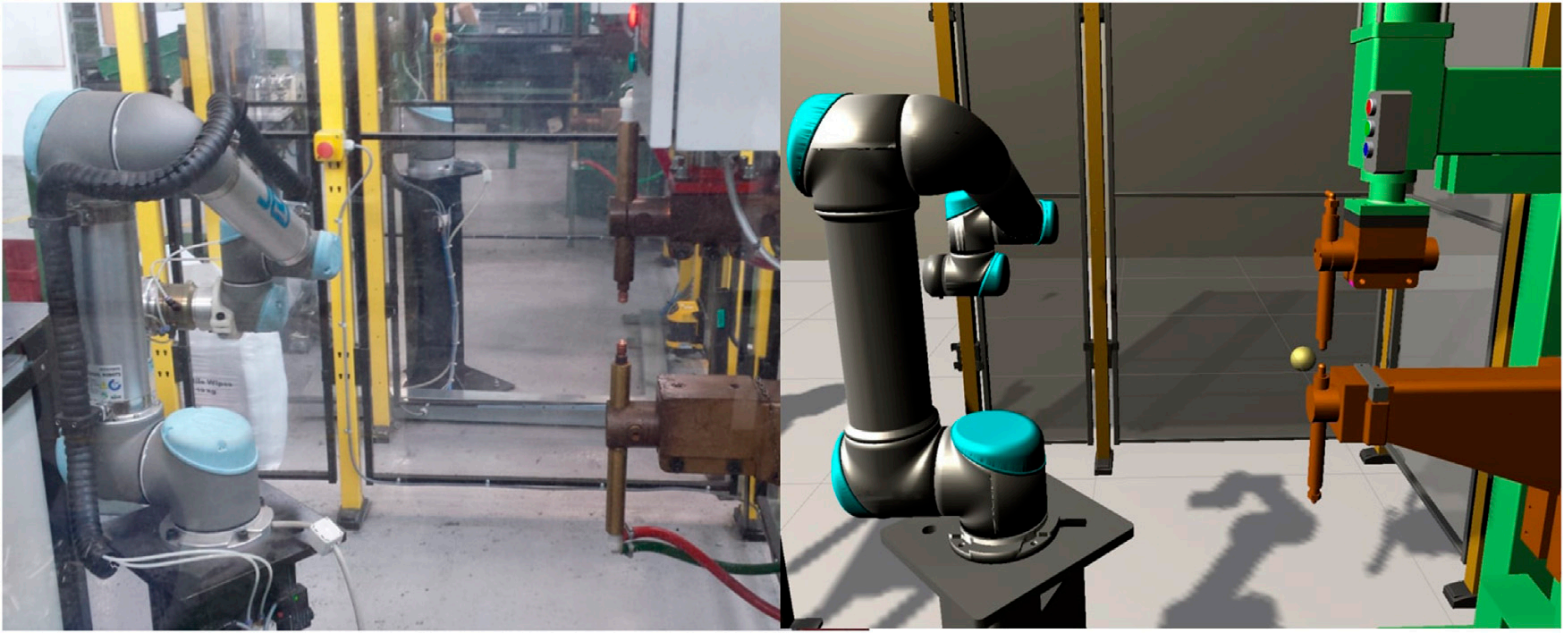
A side by side view of the physical **(left)** and digital **(right)** twins in an existing real-world industrial welding process.

The case study we examine involves the exchange of a work-piece between a human operator and a collaborative robot in order to complete a spot-welding task. The robot separates the operator from the hazardous welding machine (seen in Figure 5) with a shared handover table used to exchange assembled/welded components between the operator and robot. The robot cell is open on one side, enabling the operator to enter the cell if required. A safety LIDAR at the base of the welding machine and a light barrier at the handover table are used to monitor incursions and provide safety feedback to the system.

For our DT representation, the process is defined by a set of physical devices, namely; a robotic manipulator, a smart-spot welder, safety LIDAR, a light barrier and a human operator. An entity corresponding to each physical component is selected from the entity library and placed in the DT scenario. The exact geometry of the robot cell is measured from the physical cell or from the CAD model, and transferred to the CSI framework (as seen in Figures 1, 5). The human operator is introduced as an entity module providing a parametric autonomous avatar of the collaborating staff member.

The manipulator, smart spot-welder and sensors are physically connected to a common LAN with known static network addresses. A machine hosting ROS and the appropriate middleware for each component is introduced and connected. The network address of the ROS host is then transferred to the CSI framework in the form of a new service module targeting its address and protocol in order to create a data access point as seen in Figure 2. A visualisation of the DT and the corresponding PT at our United Kingdom partner is shown in Figure 5.

### 5.2 Assuring Safety

In this application, safety engineers, control engineers, and certification authorities are interested in enhancing the safety of the standalone PT. As mentioned in Section 4.2.3, the CSI framework presents two distinct opportunities to investigate safety assurance; as either an online interface to the physical system or as offline simulation instance of the real-world process. In this case-study we demonstrate the CSI framework using the assembled scenario DT as an offline process interface to validate an experimental safety controller ahead of deployment.

The CSI framework allows a holistic consideration of the scenario, as well as a common communication medium for the PTs to communicate with their digital counterparts, their controllers and services, and vice-versa. Presenting the data from each entity DT in a common digital space presents numerous opportunities to investigate, assess and enhance the safety of the presented process. For example, as a result of the CSI framework integration, the planned trajectory of the robot can be predicted, tested, validated, and visualised in the digital environment prior to (and in parallel with) execution in the physical space. The CSI framework may also be used to interrogate the state of the scenario and entities at any point during execution, to evaluate the state of the system and instigate risk mitigations in the process.

An often followed approach of instigating risk-mitigating changes to a scenario to prevent unsafe operating conditions is a *safety controller*. Using the CSI framework as an offline interface to the process, the design of such a controller can be evaluated and iterated.

The claim of the safety case for the robot welding cell is defined as “operator harm is highly unlikely”. The first part of the assurance task is to formally verify this claim in a conceptual model (Gleirscher and Calinescu, 2020). The second part of this task is the transfer of the verification result in the digital cobot welding cell, most practically, by validation of the concrete controller for a main use case by means of simulation-based testing (Lesage and Alexander, 2021). In the following, we discuss how the CSI framework can be utilised to carry through such a validation step. Technical details about the approaches followed for model verification and simulation-based testing are available from Gleirscher and Calinescu (2020); Lesage and Alexander (2021).

### 5.3 Controller Module Implementation

Gleirscher and Calinescu (2020) propose a tool-supported method for developing supervisory discrete-event safety controllers responsible for maintaining safety conditions in human-robot collaborations. In Gleirscher et al. (2021), a formal approach to deriving a correct *conceptual controller module* is discussed, first independent of a target execution platform and then in the context of the deployment and validation of this module on such a platform, the present CSI framework.

In our example (see Section 5.1), while the robot follows a programmed workflow, the occurrence of a hazard (e.g., the operator enters the cell during robot or welder operation) triggers the safety controller’s intervention. The controller switches the cell, particularly the robot and the welder, into an appropriate safety mode (e.g., speed and separation monitoring) and a safer task (e.g., work piece transfer instead of welding) based on the current state (including safety mode and task) of the DT environment. Once the hazard has been cleared (e.g., by interaction of the controller with the operator and the operator leaving the cell), the controller resumes the process with a relaxed safety mode (e.g., normal mode) and the original task, if possible, such that normal operation can directly continue or resume from a defined workflow position.

This conceptual controller, based on hazard analysis and risk assessment, has to be transferred to a concrete executable for evaluation and eventual deployment on the live cell. In this article, we demonstrate a deployment of this controller on the CSI framework in the form of a behaviour module. To accomplish this task, we need to 1) define the interface between the controller and the (real or digital) welding cell and 2) generate an executable controller module.

#### 5.3.1 Defining the Interface Between the Controller and the Cobot Welding Cell

An interface can be defined both syntactically and behaviourally (Broy, 2010). Following this paradigm, we first define the channels through which the controller monitors and controls (real or digital) properties of the cell and the data types of these channels (Table 1). The controller has control over the *safety mode* of the whole cell (i.e., all machines in the cell), over the *activity* of each machine, and can interact with the operator through *notifications*. Furthermore, the controller observes these properties and has access to the *robot location* and two sensors; a *range detector* and a *light barrier*.

**TABLE 1:** Syntactic interface between the safety controller and the cobot welding cell DTs.

*Property*	Channel datatype	Monitored	Controlled
Safety mode	Mode = {normal, pflim, … }	●	●
Robot location	Place = {atTable, inCell, … }	●	
Welder activity	Task = {idle, welding, … }	●	●
Robot activity	Task = {idle, exchange workpiece, … }	●	●
Range detector	Range = {far, near, close}	●	
Light barrier	Boolean	●	
Notification	AlarmSignal = {leave cell, leave workbench, … }	●	

**FIGURE 6:**
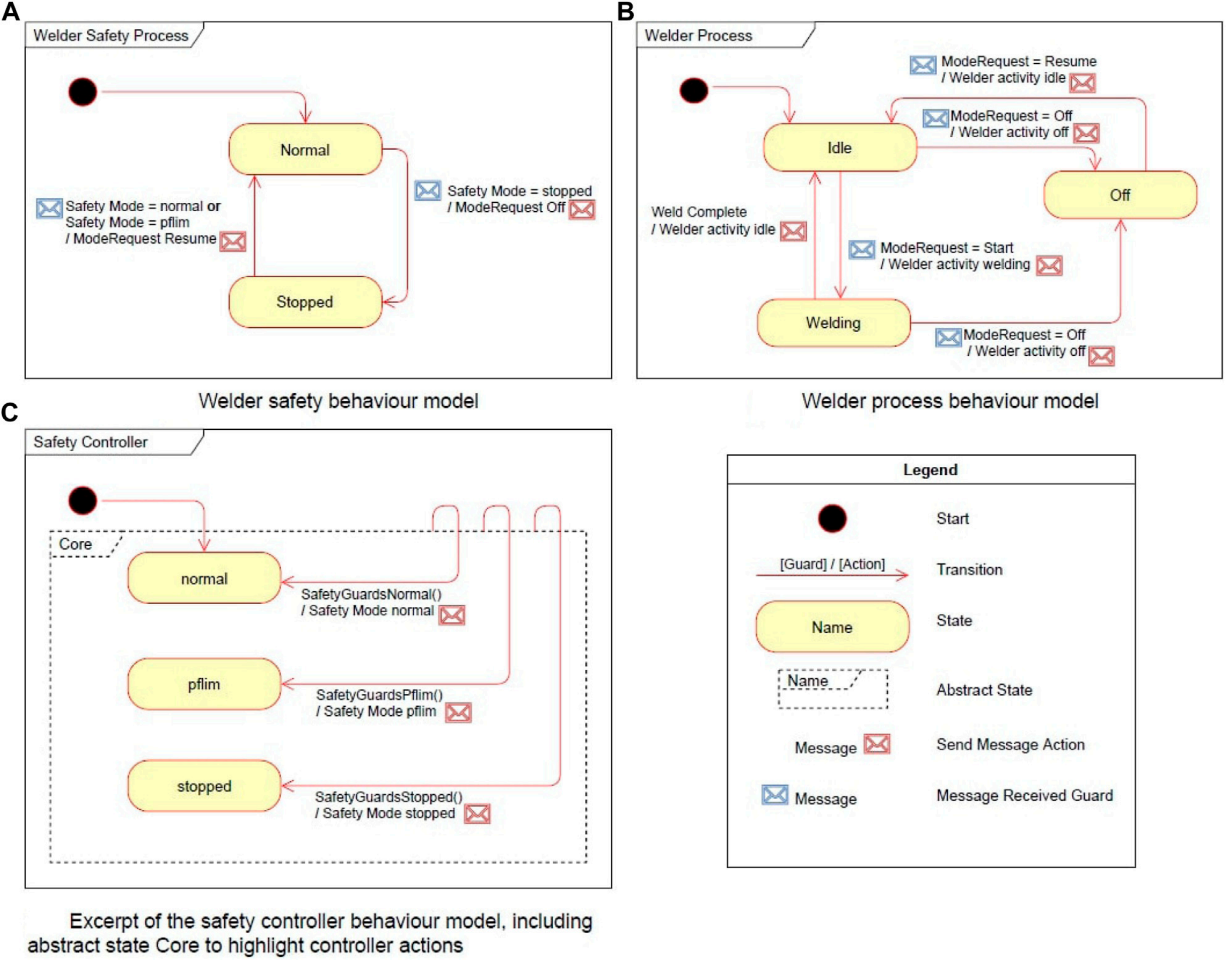
An overview of the state machines defining the behaviour of the welder in the welding cell, highlighting the interactions between the process and safety layers. An excerpt of the safety controller state machine is included to highlight the control of the safety mode and welder activity. The transitions capture both the messages sent, and the guard enabling the transition. **(A)** Welder safety behaviour model. **(B)** Welder process behaviour model. **(C)** Except of the safely controller behaviour model, including abstract state core to highlight controller actions.

Second, we define the *behaviour* of the machines under hazard-triggered supervisory control. Using state machines, we define what it means for a welder or robot to change its safety mode and task on request by the safety controller. That is, each machine needs to implement a subset of supported safety modes (e.g., normal and stopped for the welder, pflim [power and force limiting] and ssmon [speed and separation monitoring] for the robot) and a subset of tasks (e.g. idle and welding for the welder, exchange work-piece and idle for the robot). Three state machines for this behavioural scheme are exemplified in Figure 6. The dashed state “Core” in Figure 6C is an abstract state comprising the three modes normal, pflim, and stopped, meaning that the three transitions outgoing from “Core” can be triggered from each of the three modes.

#### 5.3.2 Generating and Connecting the Controller With Sensors and Actuators

**FIGURE 7:**
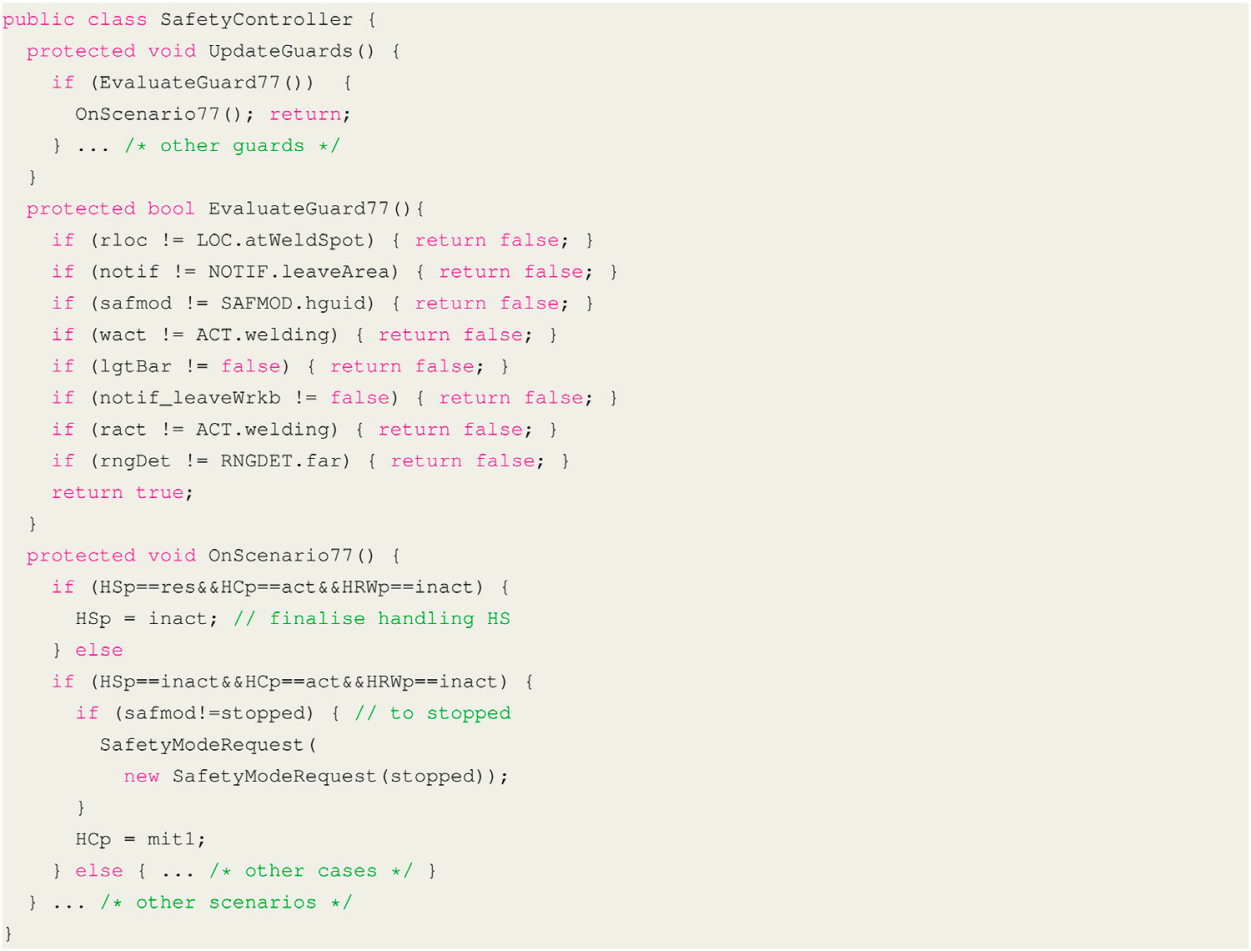
Safety controller module sample code depicting the structure of the safety controller.

**FIGURE 8:**
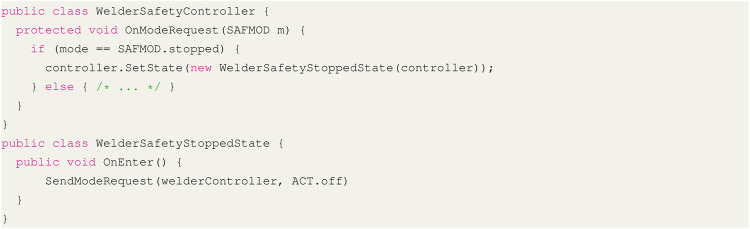
Spot welder safety mode transition sample code (Figure 6)

We implement the described interface (Table 1, Figure 6) using the infrastructure (i.e., entity and service libraries) provided by the CSI framework. Based on this interface, we derive the concrete controller from the conceptual controller module. Automatic controller synthesis, out of scope here, is done by the YAP tool in the context of probabilistic model checking and described in more detail in (Gleirscher and Calinescu, 2020; Gleirscher, 2020). The generated SafetyController (implemented in C#, Figure 7, Figure 8) is imported as a behaviour module and assigned to the robot entity module. For deployment, the generated controller is connected *via* the channels made available by the host CSI framework. As described in Section 4.2.1, an interface for communication between modules, presented in the form of message transport behaviours, allows the controller to subscribe to state updates (e.g., the location of the robot arm and end-effector) from the robot DT and event notifications from the sensor DTs (e.g., the state of the light barrier).

### 5.4 Deploying a Validation Technique as a Module

To assess the behaviour of systems modelled in the CSI framework as scenarios, in particular regarding safety aspects, we defined and evaluated an analysis technique in related works (Lesage and Alexander, 2021; Gleirscher et al., 2021). The technique uses the scenario DT as a proxy of the system PT during evaluation. The module interacts with the scenario DT to explore reasonable test cases, varying the runtime configuration of the system, and process the collected data to ensure the desired safety conditions hold in the system. Coverage metrics, supported by the module, provide an assessment of the quality of the generated test cases.

The validation technique itself is defined as an external tool, a set of Python utilities addressing various aspects of the interaction with the CSI framework. External tooling facilitates the use of offline monitoring techniques for validation, at no runtime cost, and the integration of the DT with automated test heuristics. As described in Section 4.2, the CSI framework uses open-standard formats for configuration and data export, respectively JSON and SQLite, easing the process of interacting with a scenario DTs from various languages.

In our use case, the validation needs to ensure the synthesised safety controller module, and its integration, maintain the same safety conditions required to be fulfilled by the conceptual controller module. The CSI framework allows the collection of data, in the form of execution traces, that describe the behaviour of the safety controller under defined test conditions and model assumptions. This information is key to evidencing the claim (cf. Section 5.2) that the welding cell is safe.

#### 5.4.1 Collecting and Validating Scenario Traces

The validation module needs to capture a view of the events that occur in the scenario DT to assess its behaviour. Such a trace of events can then be validated against predicates capturing important functional or safety-related properties, e.g., ensuring any ongoing weld is interrupted within an acceptable time frame when the operator enters the cell.

A behaviour module was introduced to the CSI framework as a message *snooper*. This allows for the registration of all, or a selection of, messages exchanged between entities during execution. The execution procedure of the welder safety and process controller can be seen in Figure 6A,6B respectively. The module was configured to capture the messages both to and from the controller as per Table 1, that is the state of the various entities, the commands issued by the controller, and the hazards identified by the controller. The messages collected during repeated runs of the scenario DT are then processed to build the trace of events in the system. Simple conversion rules defined for the case study map the message’s contents onto a number of time series, based on the message type, incoming channel, and emission timestamp. As an example, light barrier status messages result in a time series capturing the sensor’s state over time.

**FIGURE 9:**
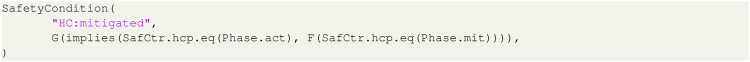
Example of a safety predicate used for verification, expressed as a temporal logic formula. It ensures that if at any time in the events trace hazard HC occurs in the work cell, as defined by the safety analysis, it is later mitigated by the safety controller. It cannot be ignored, or let to disappear unacknowledged.

All traces are then checked against a number of validation rules and any violation is reported for the user to review. Validation rules, such as safety conditions, are expressed as metric temporal logic predicates (Basin et al., 2015), exemplified in Figure 9. Such predicates can assess the ordering of events in time, ensure specific ones always hold, or that events occur within specific time windows. For our case study, all generated traces were validated against the same safety conditions used for the conceptual controller model. This validates the synthesised safety controller on the same properties that ensured the safety of the model.

#### 5.4.2 Automating Testing of the Scenario

The CSI framework supports a scenario configuration JSON, as part of P1 in Figure 4, that exposes the properties of all entities in the modelled system. As a runtime configuration file, the scenario configuration JSON allows for reasonable variations of the same scenario. The validation module exposes a thin layer to allow users to specify which configuration points they want to explore during testing. Given a test vector, a valuation of those configuration points, the validation module can generate a runtime configuration file for the CSI framework, instantiate the scenario DT, and validate the resulting trace.

**FIGURE 10:**
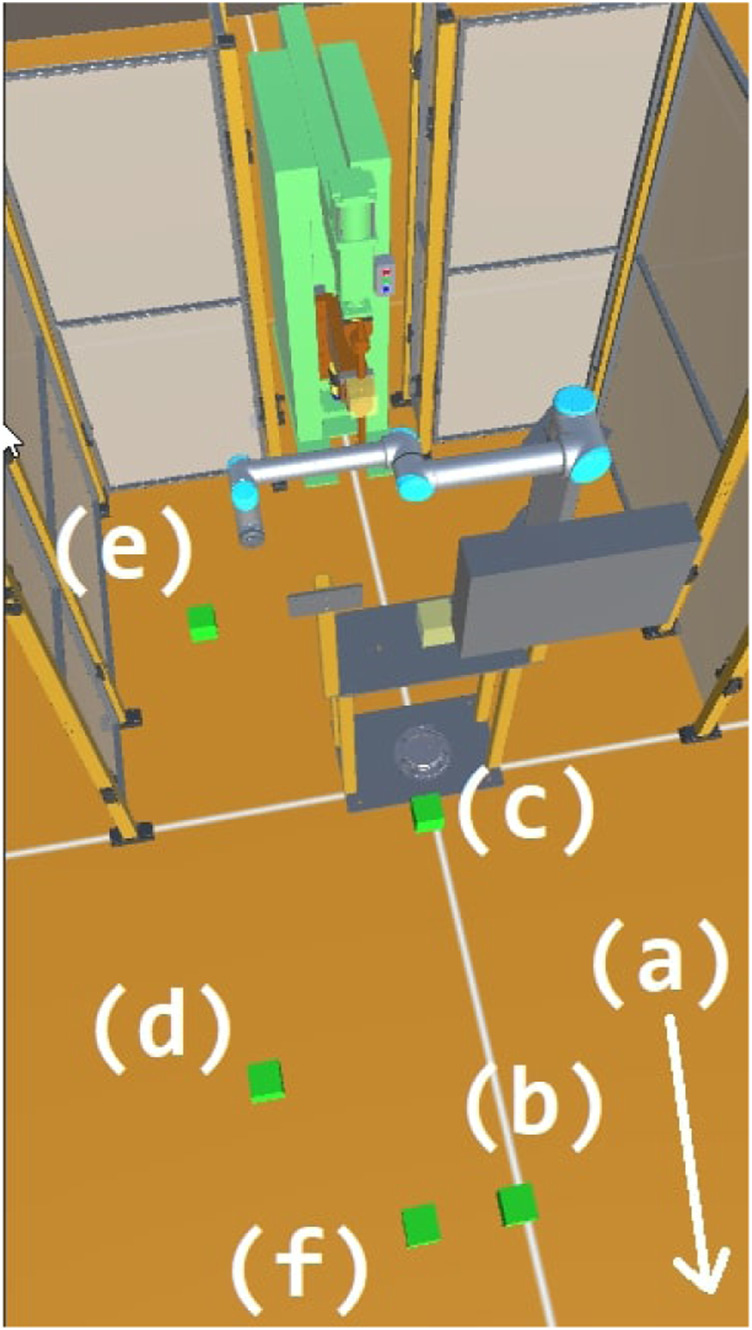
System configuration during testing, the time spent by the operator at each of the labelled waypoint is exposed as a configuration variable.

In our use case, we focus on varying the behaviour of the operator and the moment he interrupts the work in the cell. The runtime configuration includes the time spent by the operator at each waypoint of their trajectory, illustrated in Figure 10. A test vector is thus defined as 5 wait times to control when the operator would enter the cell or access the shared bench. Test vectors are randomly generated with an added constraint that the path should be completed in 20 s, allowing the cobot to complete its work even following an interruption.

The validation module relies on situation coverage to assess the performance of our testing strategy, and determine if further testing is required (Alexander et al., 2015). Situation components are defined by the DT events and their expected values. Situations can be defined from either individual components or combinations thereof, e.g. respectively observing the welder in all its activities (see Figure 8) or by, combining the Range detector and Robot location properties (see Table 1), ensuring the operator gets *close* to the welder while the robot is *at the table*. The same event traces used for validation also support the computation of the coverage achieved by a set of tests. Our coverage criterion for the controller validation ensured all sensor states in isolation were covered during testing. We also enforced that operator interference was observed at all stages of the process, from the collection of the work-piece at the shared bench to the welding of the work-piece, at either the shared handover table or within the cell.

### 5.5 Evaluation

The validation was performed using the CSI framework as a simulation-based test-bed. Upon initialisation of the scenario DT, a JSON configuration file is imported to configure entities and their behaviour to reflect to the defined test conditions. The scenario is executed and interactions between the entity modules are thus observed under variable conditions.

We performed 100 tests for the considered use case, each running for 30 real-time seconds. The whole test suite took less than 2 h to complete, including the processing and validation of generated traces, on an Intel^®^ Core i5-8250U4-core CPU at 1.8 GHz, with 8 GB RAM under the Windows 10 Home Edition operating system, build 19 042.985. No run was observed that violated the monitored safety conditions. Full coverage, based on the discussed situation-based criterion, was achieved. This ensures all hazard sources have been encountered during testing, and properly mitigated by the integrated safety controller module.

## 6 Discussion

In this article, the CSI framework was introduced as a versatile, modular tool for assembling safety-critical digital twins and the wider investigation of safety assurance in collaborative robotic processes. The framework was designed to support the evaluation of novel safety-related techniques in a wide range of scenarios, where humans and robots work together to achieve a common goal.

The CSI framework provides an environment for developing, implementing, and testing safety techniques (whether digitally or physically or both). At the core of the CSI project is the evaluation of new techniques to understand and mitigate the safety of a collaborative environment. The modular DT paradigm supports the collection of evidence for safety assurance tasks on new scenarios offline and before their deployment, as we demonstrated with the addition of a novel work-preserving safety controller in an industrial work-cell. The introduction of twin-mode switching means that digital and physical components can coexist in the scenario using variable DM, DS and DT representations; physical components can be phased into the scenario progressively, transparently replacing their digital counterparts, as confidence in the safety of the system grows and the scenario is moved online. This produces a modular environment across DM, DS and DT both in hardware and software.

The modular architecture lays the groundwork for a library of ready-for-purpose behaviours, services and entity modules. Modules can be “dropped” into the scenario from this library to quickly establish a working scenario DT. The same modularity, stemming from the framework messaging infrastructure, allows users to enrich scenarios with their own behaviours and analyses simply by defining the communication interface. The scenario constructed in Section 5.2 illustrates how critical process information can be extracted to support these use cases. This scenario was built using existing modules for the various machines and components; the safety controller and validation modules were the only extensions to the framework, respectively as a new behaviour module and interfaces for an external analysis tool.

We have created a scenario DT, based on an industrial case study, that is sufficiently accurate and provided the required evidence to demonstrate and validate a synthesised safety controller with the aforementioned modules. The approach described here paves the way for further offline safety analysis of collaborative robot processes, and a stepping-stone to full validation on physical systems. The CSI framework is agnostic to virtual or physical applications, and allows the acceleration of development using virtual resources to perform the significant safety analysis without requiring the physical resource, a critical factor in how manufacturing will deal with COVID-19 and its impact on maintaining existing and developing new processes on the shop floor. This provides a valuable tool for designers to prototype and evaluate safety schemes offline, and rapidly transfer these to physical systems with minimal effort. It also opens up exciting opportunities for development of digital techniques for safety assurance in collaborative robotics.

The CSI framework provided a safe environment for the iterative integration and revision of the synthesised safety controller, a virtual test-bed for experimentation carrying no risk for actual operators or physical assets. We used the opportunity to repeatedly test and refine the system configuration. Fine control over the scenario helped us identify and resolve issues during the synthesis of the novel safety controller and inform its design. We used this opportunity to further evaluate the system response to misuse cases where two operators interact with the cell, violating the safety controller’s design assumptions.

The process captured by the scenario DT presents an interactive model of the considered welding cell and it allows the exploration of varied, realistic configurations, all instrumented to assess the overall safety of the system. It provides credible evidence on the behaviour of the individual components, the consequences of the operator’s actions and the process as a whole. In Section 5.4, successful test results did validate the behaviour of the safety controller and, as part of our safety case (see Section 5.2), they support the claim that the considered cell is safe under the assumption that a single operator interacts with the welding cell. The misuse case evaluation and the related test cases further provide initial insight on how those assumptions could be leveraged, and further validated.

As part of the wider safety context, the presented case-study demonstrates the ability of the CSI framework to provide and expose critical process information to support multi-level scenario analyses. In Section 5.4.1, this information is used to validate the response of a novel safety control to varying conditions within a virtual cell, but could by employed to new module configurations to explore new behaviours, potential risks and scenarios.

The information exposed by CSI framework, and a given scenario DT, reaches beyond twins of the physical entities or default entity modules. This is because entity modules are limited in that they *only* embody the capabilities of their target PT. The framework however creates an interface to capture relevant information, beyond the capabilities of the PT, as a means to inform the safety of PT or of the process as a whole. This may be to support analyses with supplementary data sources for comparison, or ground truth, or even provide a placeholder for sensing gaps that might exist in the PT. These might take the form of sensory behaviour modules, that may provide metrics unavailable in the real-world. An example would be capturing the distance between the operator and the robot irrespective of the LIDAR blind spots. Such tools are key to the experimental validation of safety conditions, generated as part of the system safety analysis.

## 7 Conclusion

In this paper, we present a highly modular environment for the development of safety critical digital-twins for collaborative robotic processes. We have shown how sophisticated, multi-level, scenario DTs can be assembled to represent complex, collaborative environments with variable configurations and relationships with external hardware. This is achieved using a framework fundamentally based on modularity, allowing systems and scenarios to be constructed with ease using a library of standardised entity, service and behaviour modules. This modularity has been heavily influenced and informed by our wider safety research, and by the tools and techniques we are using to provide safety assurance.

A novel contribution of this work is in the use of modular DTs for safety research. We have shown how a scenario DT can be easily created and used to investigate and collect evidence to support safety claims made against a collaborative robotic process. This opens up exciting avenues for further research; beyond the work described in this paper we are using the framework to train safety sensing and decision-making systems, monitor for cyber-security intrusions, visualise safety information, and provide training in robotic safety assurance techniques.

Whilst COVID-19 restrictions have prevented access to physical facilities and testing on practical systems, the CSI framework has enabled us to continue our work and been shown to be capable of providing evidence to support offline safety investigations. This has been evidenced in an example case-study, in which both a novel safety controller and validation analysis are implemented using the CSI framework. The results demonstrate that the controller performed safely in 100 scenarios enacted in the DT, supporting the case for the controller’s practical deployment.

Although the work described above has been carried out virtually, the framework is agnostic in its application in physical or virtual environments; its design allows for physical entities to be connected and controlled as and when available (as we have done in other applications). Consequently, the framework allows for great flexibility in the design, testing, and deployment of safety techniques and controllers.

To be able to form conclusions about the safety of a collaborative process, the behaviours and entity (DM) modules representing real-world entities must be faithful to the real world PT. The modular design of entities and behaviours will enable future work to enhance the fidelity of specific systems to be able to make more informed safety arguments and advanced monitoring tools. This will be approached by enhancing the CSI framework’s library of existing modules and tools, or by extending the library with new module definitions. This may take the form of more realistic sensor behaviours (and limitations), operator actions or more sophisticated motion planning tools.

The CSI framework has the capability to produce diverse ecosystems of modules, processing both low-level and high-level information across a number of safety scenarios. The scalability of the CSI framework will be assessed in future work; to better understand the impacts of many of the real-world challenges associated with managing and networking systems of connected modules.

## Data Availability

The datasets presented in this study can be found in online repositories. The names of the repository/repositories and accession number(s) can be found below: All of our data, code and simulation videos are made available through our public repository https://github.com/CSI-Cobot/CSI-artefacts/tree/master/digital-twin-framework.

## Footnotes

1
https://www.sheffield.ac.uk/sheffieldrobotics/about/csi-cobot

2 All data, samples and presented data can be found on the CSI project repository: https://github.com/CSI-Cobot/CSI-artefacts

3 This is achieved using *Object Orientated Programming* (OOP) concepts of inheritance and polymorphism

4 The publisher-subscriber convention is analogous to the popular *Robotic Operating System* (ROS) by design to improve amenability (Quigley et al., 2009).
